# Experiences of health information-seeking among patients with cancer in China: a qualitative study

**DOI:** 10.3389/fpubh.2025.1584952

**Published:** 2025-08-26

**Authors:** Yanjun Wu, Shenglu Li, Yan Zhou, An Duan, Qiqi Zhuo

**Affiliations:** Department of Cancer Center, The Second Affiliated Hospital of Chongqing Medical University, Chongqing, China

**Keywords:** China, cancer, health information-seeking, information needs, qualitative research

## Abstract

**Purpose:**

Although information provision improves physical and psychological well-being, few studies have evaluated Chinese cancer patients’ information needs. Our study aimed to explore the health information-seeking experiences of Chinese cancer patients, focusing on their needs, preferences, and cultural influences. This will inform the development of culturally sensitive and patient-centered information provision strategies.

**Methods:**

Semi-structured face-to-face, in-depth interviews were conducted with 17 cancer patients. Participants were recruited in one oncology unit in China from November 2023 to February 2024. Interviews were audio-recorded, transcribed by two researchers, evaluated using conventional content analysis, and translated.

**Results:**

Four themes and eleven categories emerged from the qualitative data: passively received information (let nature take its course, maintain harmonious relationships); seeking emotional support (seeking positive stories, encouragement from healthcare professionals, family members’ involvement); different roles of information (reassuring, troublesome, difficult truths) and optimal way to obtain information (plain language, individualized, trust in the doctor most).

**Conclusion:**

The influence of culture on patients’ information needs is inevitable. In China, healthcare professionals should encourage patients with cancer to express their information needs in order to develop health information provision strategies tailored to their needs. Notably, emotional support helps maintain psychological well-being. Family members’ involvement in information-seeking progress is also an important component of emotional support. Information provision should be individualized and aligned with the patients’ information-seeking styles and individual differences. Furthermore, healthcare professionals must use plain language, provide accurate information, and correctly guide patients on online information-seeking.

## 1 Introduction

Globally, the number of cancer survivors continues to increase due to a growing and aging population, a rapid increase in new cancer cases, and advances in early detection and treatment (1–3). In 2020, there were approximately 19.3 million new cancer cases, of which half could survive for at least 10 years (3, 4). Furthermore, in 2022, there were more than 4,824,700 new cancer cases in China (5). However, it is crucial to recognize that cancer survivors have many needs throughout their cancer journey, and obtaining adequate information is one of the most frequently emphasized aspects (6–8). Ensuring that patients receive appropriate information has positively affected physiological, psychological, behavioral, and health-related quality of life (9–11). As suggested by Lazarus and Folkman’s Coping theory, individuals need information to evaluate stressful situations. Information seeking as a coping behavior can help individuals manage or maintain positive states during these situations (12, 13). Receiving a diagnosis of cancer is a stressful life event that can lead to psychological distress due to the cancer itself and subsequent treatment (9, 10). However, seeking disease-related information can help cancer survivors to make sense of their problems and feel more prepared for cancer treatment, thereby reducing anxiety and depression symptoms (9, 11, 14). Similarly, receiving disease-related information concerning diagnosis and treatment, pain management, and side-effect coping skills is essential to patient engagement (11, 15). Providing adequate information may give cancer survivors a greater capacity to cope and manage symptoms, improve their quality of life, be involved in healthcare decision-making, better communicate with families, and adhere to treatment and medical follow-up (15–17).

Information has always been one of the most frequently reported unmet needs of cancer patients from different cultural backgrounds, which is associated with anxiety, depression, distress, lower compliance, and dissatisfaction (8, 18, 19). For example, Latina cancer patients valued connection with God and enhanced spirituality as positive aspects during chemotherapy while expressing a lack of treatment and disease-related information to strengthen coping strategies, causing them to experience multiple physical and emotional stressors (20). Moreover, American cancer patients express different unmet information needs from Chinese cancer patients. Notably, they are eager to gain more common medical knowledge, etiology, second opinions, and similar experiences from online services (21). Islamic religious beliefs also allow cancer patients in Iran to overcome anxiety and stress through prayer and mystery. However, it also inhibits them from clearly stating their information needs. Consequently, this leads to a lack of information about the nature of the illness, self-care, treatment, and mental and financial, subsequently causing a series of psychological and physical problems for them (22). Additionally, not all patients want to be exposed to disease-related information. Some Asian women believe that cancer can be transmitted by talking or thinking about it. Thus, they tend to avoid exposure to any breast cancer information for fear of bringing bad luck to themselves and their families (23). Therefore, exploring survivors’ information-seeking experiences under the background of their culture is essential for providing tailored information support to address a person’s cultural beliefs and values.

The existing literature has well-documented that the information needs and seeking behaviors of cancer patients are deeply shaped by cultural norms, values, and social structures. However, these findings are rooted in cultural contexts that are different to those in China, where collectivist values, Confucian virtues (e.g., xiao, or filial piety) and taboos around death and illness play a fundamental role in healthcare interactions. This cultural uniqueness suggests that extrapolating global findings to Chinese patients is inappropriate, creating a critical gap in understanding how information needs manifest in this population. Xiao (filial piety) is a fundamental Confucian virtue emphasizing respect and a sense of obligation towards parents (24). Coupled with the family-oriented cultural context, when a Chinese individual suffers from a disease, their family members will make sacrifices and be responsible for supporting and caring for patients (25, 26). Additionally, death is a sensitive subject, and its mention is blasphemous and must be avoided in traditional Chinese culture (27). Moreover, cancer diagnosis is seen as a metaphor for death in Chinese societies, which has led to non-disclosure (28). Therefore, Chinese physicians and nurses prioritize telling the cancer diagnosis to first-degree relatives who decide whether to inform the patient of the diagnosis (28). After being the first to be informed of the cancer diagnosis, families will conceal and refuse to tell the patient a bad diagnosis or prognosis for fear of worsening the condition, bringing bad luck, and making death come sooner (29, 30). As a result, the information-seeking behavioral and information needs of informal caregivers rather than patients have been investigated (8). However, more patients have become aware of the diagnosis in recent years (31). Researchers also realize that information can empower patients to engage in medical consultation, decision-making, and health management (32). Thus, our study investigated the health information-seeking experiences of Chinese cancer survivors based on the Chinese culture. The goal was to explore the health information-seeking experiences of Chinese cancer patients within their cultural context, identify culturally specific patterns of information needs, and inform the development of targeted, culturally sensitive information provision strategies.

## 2 Methods

### 2.1 Study design

In this qualitative study, we performed a descriptive phenomenology study to describe Chinese cancer survivors’ information-seeking experiences and to explore and disclose the meaning of those experiences (33). Face-to-face, in-depth, semi-structured interviews were used to collect qualitative data (34). The narrative account method was used to reveal the meaning of information seeking experiences (35). Furthermore, the Consolidated Criteria for Reporting Qualitative Research (COREQ) checklist was used to report our study (36).

### 2.2 Setting

This study was implemented at the oncology ward of a tertiary referral comprehensive hospital in Chongqing, China. Participant recruitment and interviews were performed between November 2023 and February 2024.

### 2.3 Sampling and recruitment

Purposive sampling was used to recruit participants of diverse ages, educational backgrounds, diagnoses, and survival times. Patients were invited to participate if they: (1) had a cancer diagnosis; (2) were willing to participate; (3) were aged ≥ 18 years old; (4) were talkative and outspoken; (5) had stable condition without serious comorbidities; and (6) had disease awareness. The charge nurse responsible for recruiting participants pre-selected and introduced the potential participants to the researcher (XXX). The sample size was then determined based on the code saturation principle (37). After analyzing the data of the 15th participant, no additional new information emerged, and two more participants were invited to ensure data saturation. No participants refused to participate or withdrew halfway.

### 2.4 Data collection

Participants’ demographic data were collected before the interviews. Interviews were undertaken separately if potential participants agreed to participate and signed written informed consent. Interviews took place in the participants’ wards when they were alone. The participants’ wards were selected as the interview setting because the participants felt more comfortable there than in the department’s interview room. During the pilot interview, the department’s interview room made the participants nervous. Notably, the interviewer was a female graduate nurse (XXX) who had qualitative research experience and was not previously familiar with participants. Before the interview, each participant was given 5 min to recall their experiences regarding information-seeking. The interview guide was developed based on pilot interviews with four cancer patients. The semi-structured interview guide included ‘Can you describe your information-seeking experience?’ ‘What are your thoughts and feelings when you are seeking information?’ ‘How do you feel about the information you received?’ and ‘Is there any suggestion about information providing you want to share with me.’ Probe questions such as “What do you mean?” and “Could you tell me more about that?” were used to open the interview and obtain more detailed information. All interviews were audio-recorded, and the interviewer recorded important body language and facial expressions in notes. Each audio-record interview and memo were transcribed verbatim within 24 h. All interview transcripts were returned to participants to be verified. The 1st and 10th were re-interviewed due to being interrupted by phone calls and treatment.

### 2.5 Data analysis

Demographic variables were analyzed using descriptive statistics. The Nvivo 11 software package was used to aid in encoding interview data. Conventional content analysis was used to code themes and categories. This analysis starts with relevant research findings as guidance for initial codes and code categories directly from the text data (38). Firstly, two data analysts repeatedly reviewed interview transcripts line-by-line to understand divergent experiences and immerse themselves in the data while deriving initial semantic units (39). Secondly, the two data analysts independently identified and summarized semantic units based on the research questions, including extracting major codes, grouping similar major codes into categories, extracting latent and dominant concepts from the data, and forming more comprehensive themes. To this end, the two versions of the codebook continued to be discussed through face-to-face meetings among the co-researchers until the controversies were addressed. The final identified themes and categories were saved in a Microsoft Excel spreadsheet.

### 2.6 Rigour

We used the criteria suggested by Lincoln and Guba to enhance the validity and reliability (40). To ensure credibility, the coders had to set aside their personal experiences and perceptions and fully immerse themselves in the data. All themes and categories were derived from the data. The extracted codes were returned to the participants for confirmation. Fifteen participants regularly returned to the hospital for treatment and 2 completed treatment. Therefore, the extracted themes and categories were returned to 15 participants for confirmation, and all agreed with the interpretation. All co-researchers continuously examine and reflect during data collection, analysis, and interpretation to improve confirmability. To ensure dependability, the corresponding author, who specializes in qualitative nursing research, was involved and guided this study. After completing the analyses, all co-authors reviewed, discussed, and confirmed all interview texts, codes, and categories. To ensure transferability, the data collector spent enough time collecting detailed and accurate data until the data was saturated. We selected participants of different ages, genders, education levels, and disease diagnoses using purposive sampling. Finally, one researcher with 1 year of overseas study experience translated the themes, categories, and quotations into English. A bilingual person not involved in our study was invited to translate the English version into Chinese. Finally, all the co-authors and the bilingual person reviewed and revised some translations until the meaning was closer to the original Chinese text.

### 2.7 Ethical considerations

Research ethics approval was gained from the ethics committee of the host hospital in November 2023 (Approval no. 2023 ethical approval number 121). Written informed consent was signed by participants prior to the interview. Notably, the written informed consent provided information on the study purpose, procedures, voluntary principle, and the withdrawal of unaffected participants at any time. To protect confidentiality, informed consent signed by the participants was kept by the corresponding author and used only for ethical review. Additionally, we used numerical codes instead of the participant’s names in all diary notes, transcripts, analyses, and quotations. No identifiable information associated with the participants was asked for or recorded during the study. Furthermore, data were kept confidential by the corresponding author and were only available to the research team for research purposes.

## 3 Results

A total of 17 cancer patients aged 32–70 participated in the study, with an average age of 52.5 years. All participants were of Han nationality and married. Their duration of illness was from 3 to 48 months. The detailed demographic characteristics of participants are outlined in Table 1. The interviews lasted between 21.5 to 67.3 min, averaging 33.3 min. Four themes, including 11 categories, emerged from this qualitative research and are shown in Table 2.

**Table 1:** Characteristics of participants (*n* = 17).

Characteristics	Participants, *N* (%)
Gender	
Men	08 (47.1)
Women	09 (52.9)
Age group, y	
31–40	02 (11.8)
41–50	03 (17.6)
51–60	10 (58.8)
61–70	02 (11.8)
Education background	
Primary school	04 (23.5)
Middle school	04 (23.5)
High school	06 (35.3)
University	03 (17.7)
Religion	
None	16 (94.1)
Buddhism	01 (5.9)
Cancer type	
Rectal cancer	02 (11.8)
Colon cancer	03 (17.6)
Lung cancer	04 (23.5)
Pancreatic cancer	02 (11.8)
Nasopharyngeal carcinoma	02 (11.8)
Breast cancer	03 (17.6)
Endometrial cancer	01 (5.9)
The duration of illness (months)	
03	03 (17.6)
06	03 (17.6)
09	01 (5.9)
18	03 (17.6)
24	03 (17.6)
36	02 (11.8)
48	02 (11.8)

**Table 2:** Themes and categories of the study.

Themes	Categories
Theme 1: Passively received information	Let nature take its course
	Maintain harmonious relationships
Theme 2: Seeking emotional support	Seeking positive stories
	Encouragement from healthcare professionals
	Family members’ involvement
Theme 3: Different roles of information	Reassuring
	Troublesome
	Difficult truths
Theme 4: Optimal way to obtain information	Plain language
	Individualized
	Trust in the doctor most

### 3.1 Theme 1: passively received information

Participants are used to passively receiving information, which includes two categories: (1) let nature take its course and (2) maintain harmonious relationships. They considered that no effort could be made to change their fate, including obtaining information about the disease. Not asking for information also helps maintain a harmonious relationship with healthcare professionals.

#### 3.1.1 Let nature take its course

Some participants believed that life, aging, illness, and death are arranged by fate and that they cannot change them. Thus, knowing or not knowing disease-related information is meaningless. Accepting fate’s arrangement is the only thing they can do. A Participant said: “*Suffering from this disease is my fate; I obeyed fate’s arrangements…I never go to search for anything; knowing or not knowing makes no sense to me. Live as long as I can…I think we should accept our fate. People are going to die anyway. It is best to let nature take its course and let it develop*” (Participant 12).

#### 3.1.2 Maintain harmonious relationships

Most participants stated that no inquiry and unconditional cooperation with healthcare professionals is the best way to maintain a harmonious relationship. For them, asking for too much information may lead to distrust of healthcare professionals, anger, and deterioration of relationships due to disturbing them. A participant expressed: “*Your doctors and nurses are very busy, so I am afraid I will disturb you. If I want to know any information, I always ask my friends, relatives, or fellow patients first*” (Participant 1). Another participant expressed a similar situation: “*I came to the hospital to cooperate with your doctors and nurses, not to find fault. All I need to do is cooperate. I’m afraid that if I ask questions, the doctors will find me annoying and think I do not trust them*” (Participant 17).

### 3.2 Theme 2: seeking emotional support

All participants stated that they often seek emotional support from fellow patients, family members, and healthcare professionals to help maintain psychological well-being during the cancer journey. The qualitative data resulted in three emotional support categories associated with the participants’ experiences: (1) seeking positive stories, (2) encouragement from healthcare professionals, and (3) family members’ involvement.

#### 3.2.1 Seeking positive stories

All participants expressed that they have experience seeking positive stories from fellow patients. They believe that the real experiences of fellow patients are more convincing, and understanding their positive stories can alleviate their negative emotions and increase their confidence in treatment. A participant said: “*You need to seek information on the positive side and eliminate negative thoughts of dying in a short time. Some cancer patients can live for 7–8 years or even 10–20 years…I know a 70-year-old rectal cancer patient who has lived for about 20 years without any discomfort; why do I only have a few months? So, I became less worried and more confident. It has been more than 2 years since the illness, and I am even less worried”* (Participant 2).

#### 3.2.2 Encouragement from healthcare professionals

Some participants stated that cancer diagnosis, physical symptom burden, treatment course, and treatment side effects can make them develop negative emotions. Words of comfort, even white lies, from healthcare professionals can calm negative emotions because they believe that the comfort provided by healthcare professionals is more persuasive. One participant said: “*We are very anxious during the treatment process, so comfort really does make a difference. I remember a doctor said, “Although you have cancer, your disease is not the most serious type; now, cancer is a chronic disease; you do not have to be afraid.” I know it was a white lie, but it made me happy and more confident. Actually, one sentence from your medical staff is more effective than 10 sentences from others”* (Participant 15).

#### 3.2.3 Family members’ involvement

Most participants reported that family members’ involvement is significant for them. When they fall ill, their family members will be responsible for caring for them and seeking disease-related information. One participant said: “*I think it*’*s very important for families to be involved…My husband is always trying to get me information on these aspects. After he knows it, he will tell me. I do not want to search for it myself. My husband usually told me what could not be eaten or done and what to pay attention to”* (Participant 4).

### 3.3 Theme 3: different roles of information

When talking about their feelings about the information obtained, participants stated their experiences and triple roles of information assessed. There were three categories: (1) reassuring, (2) troublesome, and (3) difficult truths. Some participants stated that mastering information can make them feel assured, some participants experienced negative emotions such as anxiety and depression due to “too much” information, and few participants regarded information as confusing.

#### 3.3.1 Reassuring

Some patients pointed out that information is reassuring, which can help them better understand their disease status and cooperate with doctors for treatment. These patients are always dissatisfied with the information they receive and constantly search for more disease-related information through various methods. One participant explained that: “*Having a clear understanding will make me feel reassured. I have learned about this disease by doing overnight internet searches and taking trips to bookstores…I also searched a lot online, covering almost every corner…the materials I finished reading added up to hundreds of thousands, and I also took very thick notes…I am still continuously monitoring disease-related information”* (Participant 6).

#### 3.3.2 Troublesome

Some participants intend to avoid disease-related information and believe not knowing is best for them. For such patients, too much information is troublesome, which can increase their fear and anxiety, leading to a heavier psychological burden and hastened death. A participant said: “*I never search for disease-related information. As a patient, it*’*s better not to know the disease-related information. The more you know, the more troubles, and the heavier the psychological burden…sometimes people die of fear, not illness…after you know more, you will feel anxious and have more troubles and heavier ideological burdens. As long as you are free, you have to think about it. I feel very happy to have every day in a confused way”* (Participant 5).

#### 3.3.3 Difficult truths

Some participants mentioned that the internet has gradually become their primary source of information. However, the obtained information is difficult to distinguish between true and false. Consequently, false and exaggerated information increases their negative emotions. One participant expressed: “*I am accustomed to searching for information online, but the information on the internet is very scary and serious, saying that it will not be long before death after chemotherapy…I cannot tell whether the information was true or false, and I did not know what to believe”* (Participant 2).

### 3.4 Theme 4: optimal way to obtain information

Participants expressed three optimal ways to obtain information, which included three categories: (1) plain language, (2) individualized, and (3) trust in the doctor most.

#### 3.4.1 Plain language

A few participants noted that medicine terminology is too difficult to understand, resulting in them not fully understanding the information. Thus, they hope the information is provided and explained in plain language. One participant said: “*I did not quite understand the lecture. The doctor spoke too abstrusely…the stages of the disease are all English letters that I do not understand. I can only replay the video later to find out what it means. So I hope you can make it easier for patients to understand because we are amateurs. We do not know anything about medical majors”* (Participant 7).

#### 3.4.2 Individualized

Most participants reflected that different individuals had different information needs. The effects would be much better if healthcare professionals could provide information tailored to individual information needs. One participant explained: “*I think that different individuals have different education levels, personalities, and disease conditions and may have different ideals. Some people want to know a lot, while some people avoid it …some patients do not want to be instilled with too much information, while some people will feel panic and do not know what to do next if they do not know anything”* (Participant 13).

#### 3.4.3 Trust in the doctor most

Some participants expressed that doctors were the most authoritative and trusted source of information. Thus, they only want to obtain the necessary information from doctors. One participant expressed that: “*I rarely search for my illness on the network. I only trust doctors because they are the most professional, and I trust them the most. What they say is the truth…When I come to the hospital, the first to believe is the doctor. Doctors are the backbone”* (Participant 10).

## 4 Discussion

This study aimed to explore the health information-seeking experiences of Chinese cancer patients, with a specific focus on their information needs, preferences, and the influence of cultural factors, to inform the development of culturally sensitive and patient-centered information provision strategies. The findings revealed four core themes: passively receiving information, seeking emotional support, different roles of information, and optimal way to obtain information. Each theme revealed different dimensions of participants’ information needs, which were shaped by cultural norms, individual differences, and situational factors.

We found that Chinese cancer patients in this study were passive in their information-seeking experiences. The influence of traditional Chinese culture might explain this finding. For centuries, Chinese people have been deeply influenced by traditional Chinese philosophy (Confucianism, Taoism, and Buddhism) (41, 42). Taoism emphasizes that death is natural and an extension of life; Buddhism emphasizes that fate and ‘Inn’ and ‘Ko’ (cause and effect) determine health (43). Under the influence of both philosophies, cancer patients believed that their illness was a punishment from the Buddha and that their lives were determined by fate, which cannot be violated (26, 41). Therefore, patients do not care whether they receive disease information (26). Besides, the Five Relationships of Confucianism emphasize hierarchy and the absolute obedience of subordinates to superiors to maintain harmony with others (44). Moreover, patients recognize health professionals as experts and highly respect them, intending to interact and follow recommendations passively (43, 45). Thus, our patients intend to suppress their needs to avoid being defined as troublesome and distrusted, thereby maintaining harmonious relationships. Therefore, healthcare professionals should be aware that traditional culture may make Chinese cancer survivors feel unnecessary or afraid to express their needs, resulting in their information needs not being met. Therefore, healthcare professionals should encourage Chinese cancer survivors to be more proactive in seeking information and facilitate communication to help them express their information needs.

Consistent with other literature, patients in our study expressed that receiving information on emotional support from fellow patients, family members, and healthcare professionals was essential to maintaining psychological well-being (46, 47). Emotional support from fellow patients, family members, and healthcare professionals mirrors collectivist values in Chinese healthcare, where interdependence is prioritized over individual autonomy (25, 27). Patients like to learn the stories of their fellows’ survival and recovery experiences. They, believe that the best information based on individuals’ successful experiences is supportive and can give hope, which is also reported by Yli-Uotila et al. (48) Therefore, healthcare professionals can use interventions such as peer mentoring to assist patients with cancer to gain knowledge, social support, emotional assistance or practical help, and coping strategies from their peers (49). Moreover, encouragement from healthcare professionals was another facilitator for cancer survivors to maintain psychological well-being, which has been demonstrated in other studies. Raphael et al. (50) found in their cross-sectional survey that cancer survivors desired more psychological support (such as encouragement, understanding, caring, and better communication) from healthcare professionals. As suggested by Street et al. (51), our healthcare professionals need to listen, encourage, and express empathy during the information exchange process to avoid contributing to poorer experiences and unnecessary anxiety and distress for cancer patients. In our study, many cancer patients feel strongly dependent on their families to seek disease information. In the Chinese collective culture, patients are accustomed to transferring the responsibility of obtaining disease information to their family members. Likewise, families actively do everything for patients, including daily life assistance, making treatment decisions, providing disease information, monitoring treatment complications, etc., to maintain the physical and psychological well-being of their sick loved ones (25, 43, 52). Therefore, family caregivers and patients should be conceptualized as a care unit (19, 53). On the one hand, health professionals should encourage family members to participate in caring for patients within Chinese cultural contexts while encouraging patients to engage in their health care, helping them become more empowered. On the other hand, health professionals should explore and meet the information needs of family caregivers to help them better care for their sick loved ones.

Our study found that too much information was reassuring for some patients but troublesome for others. The “Blunting Hypothesis” might explain the result, emphasizing that individuals will adopt monitors or blunters information-seeking styles when threatened with threat-related cues (12). Monitors continuously search for all information about the threat, whereas blunters avoid it by distraction (12). Notably, information is a relief for monitors but a burden for blunters (26). Physical and psychological outcomes can be improved only when the information provided matches the patient’s information-seeking styles (26). When the information obtained by cancer patients does not match their information-seeking styles, it increases levels of anxiety, depression, and tension, reduces satisfaction with information provision and follow-up compliance, and is not conducive to disease prognosis (54, 55). It was also reported that some lung cancer patients expressed their right to be informed, while some patients were dissatisfied with information overload (56). Additionally, some patients in our study expressed that information should be individualized based on different educational backgrounds, personalities, disease conditions, and needs preferences. These results are congruent with other studies reporting that the provision of information should be individualized, considering different demographic factors, attitudes, needs, and information preferences (57, 58). Just as Miller et al. (54) assessed the information-seeking styles of women with an abnormal Pap smear using a scale and providing tailored telephone counseling, health professionals should provide information appropriate to the patient’s information-seeking styles. They should also consider different cultural, religious, attitudinal, and other factors to achieve individualized information provision.

As some patients have mentioned, the internet is an essential source of information, but the information on it is difficult truths. It has also been reported that cancer survivors intend to search for information online about their type and stage of cancer, risk factors, prognosis, and treatments (5). Notably, online health information-seeking positively impacts patients’ satisfaction with the consultation and care experience (59). At the same time, search engines such as Google are filled with incorrect or lack credible sources of information, resulting in higher levels of anxiety and other negative emotions (60). Therefore, doctors were the most trusted sources of disease-related information (61). As our patient says, “*I come to the hospital; the first to believe is the doctor*.” As mentioned in Sacca et al. (62), patients regarded online health information-seeking as a complementary source of information in conjunction with their interactions with doctors. Additionally, our study found that most participants lacked an adequate understanding of medical terms due to limited health literacy or low literacy and hoped that health information would be provided in plain language. As pointed out by Schultz et al. (63), the general public could not understand the medical jargon, reducing patients’ compliance with suggestions. Plain language also benefits patient-physician dialogue, improves communication behavior, and increases patient empowerment (64). Therefore, health professionals should be aware that the Internet has become a meaningful way to obtain information. As the most trusted information resource for patients, doctors should provide disease-related information to patients or assist patients in obtaining accurate online information while ensuring that the information provided is easy to understand.

## 5 Limitations

There exist some limitations that should be considered in our study. Participants were recruited from one hospital in one province, which may limit the generalizability. However, the research site is a tertiary referral comprehensive hospital that receives people with cancer from all over the country. In addition, to increase generalizability, we used purposive sampling to recruit participants with diverse demographic characteristics, which could provide rich information for the research question. This also results in the voices of ineloquent patients being ignored. However, the in-depth interviews provide data saturation, ensuring the results are more generalizable. Additionally, the nature of qualitative research limits our ability to examine variations in information needs among patients with different genders, ages, educational backgrounds, and disease severities. Given these limitations, future research should consider multicenter studies across diverse regions and inclusion of diverse populations to better extrapolate findings to all Chinese cancer patients.

## 6 Conclusion

Our qualitative study identified that Chinese cancer survivors’ diverse information preferences and needs were previously overlooked due to the influence of traditional Chinese culture. These insights collectively illuminate how cultural norms (e.g., collectivism, Confucian values of filial piety, and taboos around illness and death), individual differences, and contextual factors influence their information-seeking behaviors and needs. Specifically, the study found that Chinese cancer patients often take a passive approach to seeking information. This approach is rooted in cultural beliefs about fate and harmony and influences their willingness to express their information needs. This passivity, intertwined with cultural values, highlights the need to recognize how traditional norms may constrain active information-seeking. Additionally, emotional support derived from positive peer stories, healthcare professionals’ encouragement, and family involvement emerged as critical components of their information needs. This reflects the collectivist emphasis on interdependence in Chinese culture. These findings deepen our understanding of how cultural contexts frame not only what information patients seek but also *why* and *from whom* they seek it. Furthermore, the study identified that information plays diverse roles (reassuring, troublesome, or ambiguous) for patients, and their preferences for information acquisition (plain language, individualization, and trust in physicians) are shaped by both cultural trust in authority and individual differences in health literacy and coping styles. These insights reveal that information needs are not uniform but are dynamically shaped by a combination of cultural norms and personal traits. Based on these findings, we propose that healthcare strategies should be grounded in an understanding of these nuanced experiences. Specifically, healthcare professionals should proactively encourage patients to express their information needs and acknowledge the cultural barriers to active communication. In line with collectivist values, they should also facilitate emotional support networks that leverage peer experiences and family involvement. Information must be tailored to align with diverse information-seeking styles and delivered in plain language to accommodate limited health literacy. Additionally, since physicians are still the most trusted source, they should help patients navigate online information and avoid unreliable sources.

## Data Availability

The original contributions presented in the study are included in the article/supplementary material, further inquiries can be directed to the corresponding author.
